# Association between Extracorporeal Membrane Oxygenation (ECMO) and Mortality in the Patients with Cardiac Arrest: A Nation-Wide Population-Based Study with Propensity Score Matched Analysis

**DOI:** 10.3390/jcm9113703

**Published:** 2020-11-18

**Authors:** Su Jin Kim, Kap Su Han, Eui Jung Lee, Si Jin Lee, Ji Sung Lee, Sung Woo Lee

**Affiliations:** 1 Department of Emergency Medicine, College of Medicine, Korea University, Goryeodae-ro 73, Seongbuk-gu, Seoul 02841, Korea; icarusksj@korea.ac.kr (S.J.K.); hanks96@hanmail.net (K.S.H.); ironlyj@gmail.com (E.J.L.); reonoaz85@gmail.com (S.J.L.); 2 Clinical Research Center, Asan Medical Center, 88 Olympic-ro 43-gil, songpa-gu, Seoul 05505, Korea; totoro96a@gmail.com

**Keywords:** cardiac arrest, extracorporeal membrane oxygenation, cardiopulmonary resuscitation, mortality, hospital cost, propensity-score matching

## Abstract

We attempted to determine the impact of extracorporeal membrane oxygenation (ECMO) on short-term and long-term outcomes and find potential resource utilization differences between the ECMO and non-ECMO groups, using the National Health Insurance Service database. We selected adult patients (≥20 years old) with non-traumatic cardiac arrest from 2007 to 2015. Data on age, sex, insurance status, hospital volume, residential area urbanization, and pre-existing diseases were extracted from the database. A total of 1.5% (*n* = 3859) of 253,806 patients were categorized into the ECMO group. The ECMO-supported patients were more likely to be younger, men, more covered by national health insurance, and showed, higher usage of tertiary level and large volume hospitals, and a lower rate of pre-existing comorbidities, compared to the non-ECMO group. After propensity score-matching demographic data, hospital factors, and pre-existing diseases, the odds ratio (ORs) of the ECMO group were 0.76 (confidence interval, (CI) 0.68–0.85) for 30-day mortality and 0.66 (CI 0.58–0.79) for 1-year mortality using logistic regression. The index hospitalization was longer, and the 30-day and 1-year hospital costs were greater in the matched ECMO group. Although ECMO support needed longer hospitalization days and higher hospital costs, the ECMO support reduced the risk of 30-day and 1-year mortality compared to the non-ECMO patients.

## 1. Introduction

Extracorporeal membrane oxygenation (ECMO) is a method for temporarily providing support for heart or lung function during cardiopulmonary failure [1]. ECMO support is used in patients with refractory cardiac arrest without return of spontaneous circulation despite advanced cardiac life support, as well as in patients with cardiogenic shock, or refractory ventricular arrhythmia [2].

The use of veno-arterial ECMO, including extracorporeal cardiopulmonary resuscitation (ECPR), is rapidly increasing in adult patients [3,4,5]. As per previous reports, although age, body weight, and comorbidities of ECPR patients increased over a period, the overall survival rate was at 29%, and complications were reduced [3,4].

ECMO is a highly invasive rescue therapy, requiring significant medical resources and multi-disciplinary expertise, as well as a well-coordinated hospital system. Due to a high resource demand, the outcome may be affected by the hospital ECMO volume, patient’s economics status, and the patient selection criteria [2,6]. Survival to discharge rates following conventional cardiopulmonary resuscitation (CCPR) have been estimated at 10–20% for cardiac arrests [7,8,9]; survival to discharge rates of ECPR have been reported to be 22–36% for adult out-of-hospital cardiac arrests (OHCA) [10,11,12] and 38–46% for adult in-hospital cardiac arrest (IHCA) cases [13,14,15]. ECMO usage suggests improved survival in refractory cardiac arrest and cardiogenic shock, which are typically associated with high mortality rates [16,17]. Although ECPR seems to improve outcomes compared to CCPR, the results of many systematic reviews and meta-analysis were inconclusive due to heterogeneity, especially in OHCA cases [11,18,19,20,21]. The outcomes varied due to the use of different participant selection criteria, protocols, and strategies, according to relevant regional emergency medical services and hospital response systems [11,16,18]. Several randomized controlled trials for evaluating the benefit of ECMO support under resuscitation are in progress. However, large multicenter observational comparative studies performed in participants matched for demographic factors and comorbidities are rare, especially for analyzing the long-term outcomes and hospital costs [21].

The objective of this study was to analyze the association of ECMO support with short-term and long-term mortality in patients with cardiac arrest, based on data from the National Health Insurance Services (NHIS) program. We also compared resource usage (for resources such as multiple treatments, hospital costs, and disposition) between the ECMO and the non-ECMO groups, using a propensity score-matched analysis. This NHIS program covers almost the entire Korean population and all medical facilities in the country [22].

## 2. Methods

### 2.1. Data Source

We used cohort data from the nationally representative administrative claim database released by the National Health Insurance Sharing Service, in South Korea. The NHIS program (a unique single insurer) is a universal healthcare system administered by the government of Korea [22]. The NHIS, in which all Koreans are mandatorily registered, covers almost the entire Korean population and medical facilities in the country. It comprises of a health insurance program, which covers approximately 96% of the Korean population, and a medical aid program, which covers 3–4% of the population [23]. The database contains de-identified information on all insurance claims such as age, sex, residence, an identifier for the clinic or hospital, and type of insurance, diagnosis as coded by the International Classification of Diseases, 10th edition (ICD-10), and information on reimbursement for each medical service including medications, procedures, and patient deaths [24]. This study was approved by the Institutional Review Board of the Korea University Medical Center (#2017AN0083). This study was a retrospective study based on the de-identified administrative database, so the informed consents were waived.

### 2.2. Study Population

**Figure 1:**
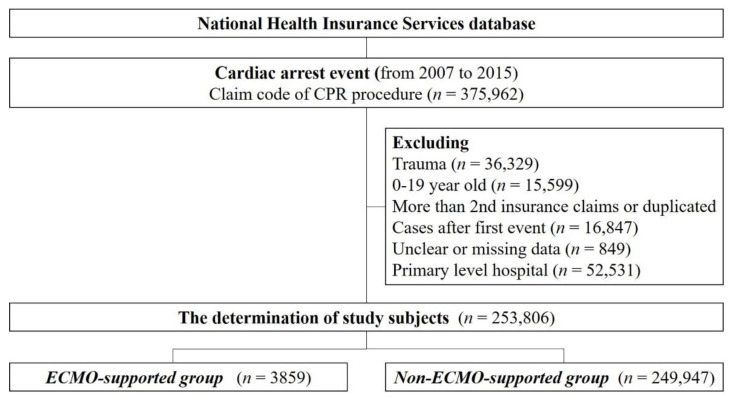
Flowchart of selection of study patients.

We identified adult patients (age ≥20 years) with the claim code for a cardiopulmonary resuscitation (CPR) procedure claim code (M5871, M5873-7), with one-year follow-up, using the NHIS claim data. All adult patients with cardiac arrest, who were admitted to the secondary or tertiary level hospital between January 2007 and December 2015, were included. Index hospitalization was defined as the first instance of hospitalization of a patient with a claim for cardiac arrest. We excluded patients below 20 years of age, those with codes from an oriental medical institute, drug store, or dentistry, and those with missing data (Figure 1). Patients who received the ECMO run were identified via the appropriate claim codes (NHI procedure codes; O1901-O1904; material codes of G5401, G5501, among others) [25]. We divided the participants into the “ECMO” and the “non-ECMO” groups.

### 2.3. Definition of Variables

We used raw data from the NHIS database to identify age, sex, residential area, type of health insurance at index hospitalization, pre-existing comorbidities (differentiated by diagnostic codes at the medical institute before index hospitalization), and the Charlson Comorbidity Index (CCI) of the participants, by referring to the diagnostic codes [26] and hospital information. The level of hospital is classified by the Ministry of Health and Welfare based on the hospital’s level of medical service, function of medical care and training, human resources, facilities, etc. Tertiary-level hospitals include more than 20 professional departments with a resident training function, while secondary-level hospitals include a minimum of 100 beds with seven to nine professional departments. The urbanization levels of the participants’ residential areas were classified on the basis of the geographical region of the administrative divisions.

A pre-existing disease was classified [27] and determined when a diagnostic code was recorded at least twice within one year during visits to clinics, or when a patient had one or more hospitalizations within two years before index hospitalization. Further, we extracted details regarding specific treatments including defibrillation, percutaneous coronary intervention (PCI), coronary angiography (CAG), coronary artery bypass graft (CABG), implantable cardioverter defibrillator (ICD) or pacemaker, continuous renal replacement therapy (CRRT), hemodialysis, electroencephalography (EEG), targeted temperature management (TTM), and prescription medication information from the records of reimbursements for each medical service during index hospitalization. The estimated total costs, length of stay, and post-hospitalization disposition until one-year follow up from index hospitalization were also extracted. The exchange rate was assumed to be KRW 1155 per Unites States Dollar (USD) 1.

### 2.4. Study Outcome

The primary outcome was mortality rate within 30 days (short-term), and one year (long-term) of the index date. Secondary outcomes were total short-term hospital costs during the acute care period (within 30 days of index date) and long-term costs (from 31 days to within one year of the index date).

### 2.5. Statistical Analysis

Demographic data were described using proportions for categorical variables and mean with standard deviations (SD), and median with interquartile range (IQR) for continuous variables. For the analysis of effectiveness of ECMO vs. non-ECMO, we used the propensity score (PS) matching method. The propensity scores were estimated without regard to outcomes using multiple logistic regression analysis. A full non-parsimonious model was developed that included all variables shown in Table 1; Table 2 (age, sex, insurance type, urbanization level, level of hospital, volume of hospital, admission route, cancer, ischemic stroke, hemorrhagic stroke, myocardial infarction, angina, heart failure, arrythmia, hypertension (with medication), diabetes mellitus (with medication), lipidemia, pulmonary disease, chronic renal failure). A propensity score matching was performed to control selection biases and to determine causal effect of ECMO groups on outcomes. Using the Greedy Match algorithm, we created propensity score–matched pairs without replacement (a 1:1 match). After propensity score matches were generated, balance in baseline covariates of two groups were assessed using absolute standardized differences (ASDs). For all variables, ASDs less than 0.1 were considered to represent a small, standardized difference [28]. The outcomes were compared by use of logistic regression analysis with generalized estimating equation (GEE) methods with robust standard errors that accounted for the clustering of matched pairs. In Table 3, Table 4 and Table 5, we used McNemar’s test, Bowker’s symmetry test, and linear regression with generalized estimating equations method accounting for the clustering of matched pairs. All *p*-values were two-sided with a significance threshold of *p* < 0.05. All statistical analyses were performed using SAS ver. 9.4 (SAS Institute, Cary, NC, USA).

## 3. Results

We identified 253,806 patients with cardiac arrest; the ECMO and the non-ECMO groups accounted for 98.5% and 1.5% of the population, respectively. Furthermore, 61.6% and 12.2% of patients were male, and rural residents, respectively. Additionally, 13.3% of the patients were insured by the medical aid program, and 18.2% and 60.35% of the patients used lower-capacity (<300 beds) and secondary level hospitals, respectively (Table 1). Of the total population, 77.8% were admitted through the emergency room (ER).

### 3.1. Comparison of Demographic Characteristics, Hospital-Related Factors, and Pre-Existing Diseases, before PS-Matching

The ECMO group showed a lower age, a higher proportion of male patients, better national health insurance coverage, and a higher usage of tertiary level and high-capacity hospitals, as compared to the non-ECMO group. The rate of admission through ER was lower in the ECMO group than in the non-ECMO group (Table 1).

Compared to the non-ECMO group, the ECMO group reported a significantly lower number of patients with chronic pre-existing diseases such as ischemic stroke, chronic respiratory disease, chronic renal failure, and liver cirrhosis. The ECMO group also had a higher incidence of cardiovascular diseases like coronary artery disease than did the non-ECMO group (Table 2). Before PS-matching, 30-day, 6-month, and one-year mortality rates were 83.6%, 90.4%, and 91.3%, respectively, in the non-ECMO group; these rates were relatively higher in ECMO group, as shown in Table 2. After PS-matching, 30-day, 6-month, and one-year mortality rates were 80.5%, 87.7%, and 88.7%, respectively, in the non-ECMO group, and 75.1%, 81.5%, and 82.2%, respectively, in the ECMO group (Table 2).

### 3.2. Comparison of Provided Treatments in both Groups after PS-Matching

After all demographic characteristics, hospital-related factors, and pre-existing diseases were matched, data showed that defibrillation was more frequently administered to the mECMO (PS-matched ECMO) group (63.7%) than to the mNon-ECMO (PS-matched non-ECMO) group (33.4%). Amiodarone, atropine, and CAG were more frequently provided to the mECMO group than to the mNon-ECMO group. Specific treatments and procedures, including PCI, CABG, CRRT, and TTM, were more commonly administered in the mECMO group (Table 3).

### 3.3. Adjusted Odds Ratio of ECMO for 30-Days, 6-Month, and One-Year Mortality

Adjusted odds ratios (aORs) of ECMO were 0.73 (95% CI 0.68–0.79) for 30-day mortality, 0.66 (95% CI 0.61–0.72) for 6-month mortality, and 0.63 (95% CI 0.58–0.69) for one-year mortality in the entire population, before PS-matching. After adjusting for age group, sex, insurance status, level of hospital, hospital volume, residential area, admission route, urbanization, and pre-existing diseases, ECMO support was found to be negatively associated with 30-day, 6-month, and one-year mortality, exhibiting aORs [95% CIs] of 0.76 [0.68–0.85], 0.695 [0.61–0.79], and 0.66 [0.58–0.75], respectively, after PS-matching (Table 4).

### 3.4. Comparison of Length of Stay, Hospital Costs, and Disposition after PS Matching

The median hospital stay was 6 (2–17) days in the mECMO group and 3 (1–13) days in the mNon-ECMO group. The median intensive care unit (ICU) stay during index hospitalization was 4 (2–12) days in both groups. The median short-term hospital costs were USD 15,117 for the mECMO group and USD 2157 for the mNon-ECMO group. Long-term hospital costs were higher for the mECMO group (USD 20,324) than for the mNon-ECMO group (USD 12,780), even though there was no difference between groups pertaining to continued admission and readmission rates after discharge within the one-year follow-up period (Table 5).

## 4. Discussion

This nationwide representative study resulted in several important findings. First, 1.5% of the patients with non-traumatic cardiac arrest were supported by ECMO. The ECMO-supported patients were more likely to be younger, men, covered by national health insurance, and showed a higher usage of higher-level and higher-capacity hospitals. Second, compared with the non-ECMO group, the ECMO group showed less comorbidities except for coronary artery disease. Third, administration of advanced therapies such as CAG, PCI, CRRT, and TTM was more prevalent in the mECMO group. Fourth, hospital and medical factors-adjusted mortality was lower in the mECMO group after propensity score-matched analysis. Last, compared to that in the mNon-ECMO group, duration of hospitalization was longer and the median short-term hospital cost was six to seven times higher in the mECMO group; however, median long-term hospital cost was 1.6 times higher in the mECMO group than in mNon-ECMO group, after PS-matching.

The rate of ECMO support in patients with cardiac arrest or cardiogenic shock is increasing. In this regard, risk-adjusted survival to discharge is being maintained, despite a broadening of patient selection criteria to include older age, multiple comorbidities, and OHCA cases [3,4]. Advancements in technology such as more accessible percutaneous cannulation and increased portability of ECMO circuits have allowed for the expansion of ECMO support in emergency situations like cardiac arrest and may lead to improved outcomes. However, outcomes of ECMO support in cardiac arrest are poor, compared with those of ECMO support in other causative conditions such as respiratory failure or post-cardiotomy.

Multiple observational studies and meta-analyses have demonstrated a better outcome in the ECMO-supported patients, depending on the location of arrest [12,18,29,30], but the benefit of ECPR on outcome is still controversial in OHCA and IHCA cases, after adjusting for age and comorbidities, among other factors [11,16,21,31]. Moreover, large cohort studies and randomized controlled trials for comparing ECMO with CCPR are lacking. In our study, based on a nation-wide database, the ECMO group accounted for 1.5% of cardiac arrest patients, and showed a difference of 9–10% before matching, and of 6–7% in 30-day, 6-months and one-year mortality rates after propensity-matched analysis of demographic characteristics, hospital, and medical factors (Table A1). Our study demonstrated that ECMO support was associated with lower mortality and reduced the risk of mortality by 24–34% up to one year after matching and adjusting covariates. A meta-analysis by Ouweneel et al. also showed that ECPR was more beneficial compared with CCPR and revealed risk differences of 14% and 13% in short-term survival and long-term survival rates, respectively, in propensity-matched studies [16]. Other propensity-matched studies showed similar results with better survival rates of 15–37.5% at 3–6 months and 20–22% at one year after arrest for ECPR, compared to survival rates of 8–13% for CCPR in patients with OHCA or IHCA [12,29,30,32,33].

However, Patel et al. demonstrated that ECMO-supported patients, 2.3% of adults hospitalized with cardiac arrest, showed a similar mortality rate of 60% in the ECMO-supported and non-supported patients using inpatient administrative database [34]. After adjusting for covariates, the presence of ECMO was associated with higher rates of in-hospital mortality [34]. These results are not in agreement with our results, which may be due to different criteria of selecting the patients with ECMO support, various ways of ECMO management, regional variations in population characteristics, comorbidities, health insurance system, and healthcare culture. Moreover, the higher rate of therapeutic hypothermia in the ECMO support group may influence the better outcome in our results, while the study of Patel et al. did not show any difference of the hypothermia implementation rate in both groups [34].

ECMO support was more likely to be administered to younger men, with comorbidities of coronary artery disease, and who were admitted through the emergency room (ER). Patients with a history of cerebrovascular disease, chronic pulmonary disease, and chronic renal failure, were less likely to receive ECMO support as per the un-matched analysis. Other reports showed a higher proportion of heart failure, and a lower proportion of hypertension and DM in the ECMO group compared to the non-ECMO group [34,35]; however, there were no significant differences between both groups in our results. Patel et al. reported that the ECMO group showed a lower rate of chronic kidney disease, similar to our results in the pre-matched analysis [34]. A longer resuscitation duration, a renal hypoperfusion due to circulatory shock that needs vasopressor administration, hemodynamic fluctuations that alter renal blood flow, systemic inflammation and a hypercoagulable state, and hemolysis from blood exposure to artificial surfaces of ECMO support, may lead to acute kidney injury [36,37] Although the clinical information, including resuscitation duration, could not be revealed, the higher rate of CRRT of 42.9% in the mECMO group was shown, similar to 46.0% in the ECMO group of a meta-analysis study [38].

ECMO-supported patients tended to use higher level and higher capacity hospitals, were more covered by the national health insurance plan, and were more urban residents in the pre-matched analysis. These results are in agreement with those of other studies [34,35]. A lack of adequate insurance cover or economic status may influence access to healthcare or delivery of services like ECMO support after admission [39,40].

Extracorporeal life support in cardiac arrest is a highly invasive procedure and acts as a bridge to provide more time and allowance for confirming etiology of arrest and for maintaining organ perfusion. ECMO needs numerous medical resources, multi-disciplinary cooperation, and a well-coordinated hospital system, which needs to be accessed in a limited time; it is also associated with high medical expenses [2,41]. ECMO support presents societal burden as well as ethical burden to the patient’s family. Thus, effective resource utilization and cost-effectiveness need to be evaluated for optimal long-term outcomes and reduced burden. Identifying differences in short-term and long-term costs, and in mortality rates, based on ECMO support usage can help guide decisions on allocating resources and in providing cost-effective therapy.

In our study, the short-term and long-term costs were 3.8 times (USD 19,018) and 1.4 times (USD 30,400) higher, respectively, in the matched ECMO group (with reducing the risk of mortality of 24–34%) than in the matched non-ECMO group. A previous single-center study suggested that the cost per extra quality adjusted life years (QALY) was USD 56,000 in the United States of America [42], and several studies reported USD 11,000–29,500 or EUR 11,000–15,000 per extra QALY in ECMO-supported patients with cardiac arrests [43,44,45]. Although most studies showed inconsistent results due to differences in health insurance systems, national cost effectiveness thresholds or the Willingness-To-Pay thresholds, and national income for medical intervention, studies reported that ECMO support can be considered a cost-effective treatment. ECMO support for cardiogenic shock or cardiac arrest was associated with a higher mortality rate and lower total hospital costs, compared with ECMO support for respiratory failure or transplantation [46].

A majority of the costs of ECMO-supported patients may be determined by the length of stay, and other associated expensive procedures Our results also evidenced that the matched ECMO group showed more hospitalization days and receipt of a higher number of provided procedures. Although ECMO support needed longer hospitalization days and higher hospital costs, the reduced risk of 30-day and 1-year mortality was shown in the ECMO-supported patients.

One-year non-survivors of the ECMO group were more likely to be elderly women, covered by medical aid program, with high CCI (more than 2), to have comorbidities, such as ischemic stroke, chronic renal failure, and heart failure, in line with other studies [47,48] (Table A2).

### Study Limitations

This study has several limitations. First, since our data were based on the administrative insurance claim database, resuscitation-related variables, clinical and physiologic factors including laboratory findings, which may be representative of severity of disease, could not be analyzed

The national administrative insurance database is comprehensive, but does not include the important clinical information such as witnessed events, initial rhythm, correctable cause, CPR duration, hemodynamic data, laboratory data, management protocols, and so on. Moreover, the data on cerebral performance category at discharge were not available in this database.

In-hospital variables were obtained using secondary diagnostic codes and operational procedure codes, which lack the detailed clinical information like the intensive care and ECMO management protocol recorded during index hospitalization. The lack of clinical information in relation to time flow, such as procedure, medication use, and laboratory values, may lead to not excluding the effect of confounders. Thus, the scope for establishing a causal relationship between the clinical characteristics and outcomes is limited. Additionally, unidentified confounders that may affect the outcomes cannot be ruled out.

Second, we included only pre-existing factors, such as patients’ characteristics, hospital factors, and pre-existing comorbidities, before the index hospitalization or at the initiation of the index hospitalization, excluding the treatments in the ECMO supported group and the non-ECMO supported group, for the PS matching analysis. Extracorporeal life support in cardiac arrest acts as the bridge to treatment and recovery by maintaining organ perfusion and the ECMO support may give the chance of treatments, such as PCI, for correcting etiologies. We did not include unavailable resuscitation-related variables in our database, and confounding variables such as the performed defibrillation and treatments, for the PS matching analysis. Thus, these results may not be generalizable and confirming due to the limitations from the administrative database.

Third, since we included both IHCA and OHCA patients, the location of arrest could not be determined. Moreover, data regarding survival with functional recovery among hospitalized OHCA patients were not available in the NHIS database.

Fourth, the procedure codes for ECMO did not differentiate between veno-arterial or veno-venous EMCO modes, despite we identified the arterial cannula and catheter type. The ECMO support in the patients with cardiac arrest needs veno-arterial ECMO mode, the mixed cases with veno-venous mode can lead to overestimate the survival benefit and longer hospitalization days in the ECMO group [5]. Moreover, there might be the various ECMO management protocol, such as patient selection criteria, ways to address complications, and differences of the expertise of multidisciplinary team among hospitals [2,21]. These differences could influence the outcome; however, such information could not be analyzed due to the limitations of the administrative claim data.

Fifth, resource usage data were available only for the entire hospital admission, so these data were not representative of ECMO-specific resource use. Hospitalization costs attributed directly to ECMO could not be delineated in this study. We further could not identify ECMO support utilization during the cardiac arrest or during the subsequent shock.

Last, despite the advantages of using nationally representative in-hospital healthcare insurance data on an individual level regarding information, insurance claim data is subject to errors related to coding and omission of costs; additionally, hospital costs represent hospital billing, and not actual expenditures [49]. As non-claim data, which may have included non-standard medications and services was not covered by the NHIS, total hospital costs without non-claim data, may have been underestimated. Hospital costs also did not include information on other types of chronic supportive care for outpatient rehabilitation, nursing homes, or indirect costs of emergency medical service system. We did not measure the cost per survivor per QALY gained, it is difficult to compare our results with results of other studies. Comparability with conditions in other countries may be limited due to differences in healthcare insurance systems and cultural factors.

## 5. Conclusions

ECMO support showed a negative association with short-term mortality and long-term mortality for one year, based on a propensity score-matched analysis for hospital-related and comorbid factors. Although ECMO support resulted in longer hospitalization days and was associated with higher total short-term and long-term hospital costs, and the risk of mortality was reduced by 24–34%, compared to that in the non-ECMO-supported patients. Because this study was based on an administrative claim database, it was not possible to infer causation. Thus, a further large, registry-based study is needed.

## Figures and Tables

**Table 1:** Characteristics and hospitalization-related factors of cardiac arrest patients according to application of extracorporeal membrane oxygenation.

	Before Propensity Score-Matched Analysis					After Propensity Score-Matched Analysis			
	Total	Non-ECMO *	ECMO	*p*-Value	ASD *	Total	Non-ECMO	ECMO	ASD *
Age, years				<0.0001 ^a^	0.4492				0.0077
Mean ± SD ^†^	66.4 ± 14.8	66.5 ± 14.8	59.8 ± 14.9			59.7 ± 15.0	59.7 ± 15.1	59.8 ± 14.9	
Median (IQR ^‡^)	69.0 (56.0–78.0)	69.0 (56.0–78.0)	61.0 (50.0–72.0)			61.0 (50.0–72.0)	61.0 (49.0–72.0)	61.0 (50.0–72.0)	
Sex, *n* (%)				<0.0001 ^b^	0.1372				0.0141
Female	97,569 (38.4)	96,334 (38.5)	1235 (32.0)			2421 (31.6)	1198 (31.3)	1223 (31.9)	
Male	156,237 (61.6)	153,613 (61.5)	2624 (68.0)			5231 (68.4)	2628 (68.7)	2603 (68.0)	
Insurance type, *n* (%)				<0.0001 ^b^	0.3019				0.0148
National health insurance	219,883 (86.7)	216,214 (86.5)	3669 (95.2)			7290 (95.3)	3651 (95.4)	3639 (95.1)	
Medical aid	33,811 (13.3)	33,624 (13.5)	187 (4.9)			362 (4.7)	175 (4.6)	187 (4.9)	
Urbanization level, *n* (%)				<0.0001 ^b^	0.0740				0.0168
Urban	222,381 (87.9)	218,930 (87.8)	3451 (90.1)			6915 (90.4)	3467 (90.6)	3448 (90.1)	
Rural	30,769 (12.2)	30,391 (12.2)	378 (9.9)			737 (9.6)	359 (9.4)	378 (9.9)	
Level of Hospital, *n* (%)				<0.0001 ^c^	0.5684				0.0033
Tertiary	100,823 (39.7)	98,253 (39.3)	2570 (66.6)			5098 (66.6)	2546 (66.5)	2552 (66.7)	
Secondary	152,983 (60.3)	151,694 (60.7)	1289 (33.4)			2554 (33.4)	1280 (33.5)	1274 (33.3)	
Volume of hospital (Beds), *n* (%)			<0.0001 ^c^	0.6228				0.0555
<300	46,143 (18.2)	45,988 (18.4)	155 (4.0)			281 (3.7)	129 (3.4)	152 (3.9)	
300–499	54,905 (21.6)	54,442 (21.8)	463 (12.0)			902 (11.8)	443 (11.6)	459 (12.0)	
500–799	99,652 (39.3)	97,921 (39.2)	1731 (44.9)			3488 (45.6)	1769 (46.2)	1719 (44.9)	
>800	53,106 (20.9)	51,596 (20.6)	1510 (39.1)			2981 (38.9)	1485 (38.8)	1496 (39.1)	
Admission route (ER ^§^), *n* (%)	197,414 (77.8)	194,637 (77.9)	2777 (71.9)	<0.0001 ^b^	0.1366	5627 (73.5)	2871 (75.0)	2756 (72.0)	0.0682
Total, *n* (%)	253,806 (100)	249,947 (100)	3859 (100)			7652 (100)	3826 (100)	3826 (100)	

^a^ student’s t-test, ^b^ Fisher’s exact test, ^c^ chi-square test. ASD *, Absolute standardized differences; SD ^†^, standard deviation; IQR ^‡^, interquartile range; ER ^§^, Emergency room. * Absolute standardized difference (ASD) of >0.1 is considered meaningful.

**Table 2:** Pre-existing diseases of the patients with cardiac arrest according to extracorporeal membrane oxygenation.

	Before Propensity Score Matched Analysis					After Propensity Score Matched Analysis			
*n* (%)	Total	Non-ECMO	ECMO	*p*-Value	ASD *	Total	Non-ECMO	ECMO	ASD *
Cancer	50,084 (19.7)	49,706 (19.9)	378 (9.8)	<0.0001 ^a^	0.2868	750 (9.8)	374 (9.8)	376 (9.8)	0.0018
Ischemic Stroke	46,543 (18.3)	46,096 (18.4)	447 (11.6)	<0.0001 ^a^	0.1929	876 (11.5)	430 (11.2)	446 (11.7)	0.0131
Hemorrhagic Stroke	7904 (3.1)	7858 (3.1)	46 (1.2)	<0.0001 ^a^	0.1343	90 (1.2)	44 (1.2)	46 (1.2)	0.0048
Myocardial infarction	10,420 (4.1)	10,161 (4.1)	259 (6.7)	<0.0001 ^a^	0.1174	492 (6.4)	234 (6.1)	258 (6.7)	0.0256
Angina	40,540 (15.9)	39,688 (15.9)	852 (22.1)	<0.0001 ^a^	0.1586	1609 (21.0)	764 (19.9)	845 (22.1)	0.0520
Heart failure	29,198 (11.5)	28,803 (11.5)	395 (10.2)	0.0127 ^a^	0.0414	695 (9.1)	304 (7.9)	391 (10.2)	0.0792
Arrhythmia	25,676 (10.1)	25,329 (10.1)	347 (8.9)	0.0192 ^a^	0.0388	626 (8.2)	283 (7.4)	343 (8.9)	0.0572
HTN ^†^ + medication	104,358 (41.1)	102,711 (41.1)	1647 (42.7)	0.0479 ^a^	0.0322	3255 (42.5)	1617 (42.3)	1638 (42.8)	0.0111
DM ^‡^ + medication	51,998 (20.5)	51,134 (20.5)	864 (22.4)	0.0036 ^a^	0.0471	1708 (22.3)	849 (22.2)	859 (22.5)	0.0063
Lipidemia	71,182 (28.1)	69,781 (27.9)	1401 (36.3)	<0.0001 ^a^	0.1803	2708 (35.4)	1316 (34.4)	1392 (36.4)	0.0416
Chronic Pulmonary disease	77,667 (30.6)	76,861 (30.8)	806 (20.9)	<0.0001 ^a^	0.2269	1559 (20.4)	762 (19.9)	797 (20.8)	0.0227
Chronic Renal Failure	24,519 (9.7)	24,275 (9.7)	244 (6.3)	<0.0001 ^a^	0.125	458 (5.9)	215 (5.6)	243 (6.4)	0.0309

^a^ Fisher’s exact test; HTN ^†^, hypertension; DM ^‡^, diabetes mellitus; ASD *, Absolute Standardized Differences.

**Table 3:** Medications and procedures in the patients with arrest after propensity score matching.

*N* (%)	Total	mNon-ECMO	mECMO	*p*-Value
Defibrillation	3692 (48.3)	1279 (33.4)	2413 (63.1)	<0.0001 ^a^
Epinephrine	7411 (96.9)	3631 (94.9)	3780 (98.8)	<0.0001 ^a^
Mean ± SD	9.2 ± 21.6	5.6 ± 8.1	12.6 ± 28.8	<0.0001 ^b^
Median (IQR)	4.0 (2.0–11.0)	2.0 (1.0–7.0)	6.0 (2.0–16.0)	<0.0001 ^b^
Amiodarone	3025 (39.5)	830 (21.7)	2195 (57.4)	<0.0001 ^a^
Atropine	5391 (70.5)	2505 (65.5)	2886 (75.4)	<0.0001 ^a^
Mean ± SD	3.9 ± 5.6	3.6 ± 5.8 4.1 ± 5.4	4.1 ± 5.4	0.0075 ^b^
Median (IQR)	2.0 (1.0–4.0)	2.0 (1.0–4.0)	2.0 (1.0–5.0)	
CAG *	2803 (36.6)	433 (11.3)	2370 (61.9)	<0.0001 ^a^
PCI ^†^	1933 (25.3)	257 (6.7)	1676 (43.8)	<0.0001 ^a^
CABG ^‡^	157 (2.1)	30 (0.8)	127 (3.3)	<0.0001 ^a^
ICD ^||^	44 (0.6)	16 (0.4)	28 (0.7)	0.0641 ^a^
Pacing	930 (12.2)	186 (4.9)	744 (19.5)	<0.0001 ^a^
ETCO2 ^#^	802 (10.5)	322 (8.4)	480 (12.6)	<0.0001 ^a^
CRRT **	2079 (27.2)	435 (11.4)	1644 (42.9)	<0.0001 ^a^
Hemodialysis	269 (3.5)	103 (2.7)	166 (4.3)	<0.0001 ^a^
Brain CT	1837 (24.0)	987 (25.8)	850 (22.2)	0.0002 ^a^
Brain MRI	507 (6.6)	257 (6.7)	250 (6.5)	0.7481 ^a^
EEG ^¶^	933 (12.2)	285 (7.5)	648 (16.9)	<0.0001 ^a^
Therapeutic hypothermia	354 (4.6)	74 (1.9)	280 (7.3)	<0.0001 ^a^

CAG *, Coronary angiography; PCI ^†^, Percutaneous coronary intervention; CABG ^‡^, Coronary artery bypass graft; ICD ^||^, Implanted cardioverter-defibrillator; ETCO2 ^#^, End-tidal carbon dioxide; CRRT **, Continuous renal replacement therapy; EEG ^¶^, Electroencephalography. ^a^ McNemar’s test. ^b^ linear regression with generalized estimating equations method.

**Table 4:** Adjusted odds ratio of ECMO support for 30-days, 6-months, one- year mortality after propensity score matching.

	Crude Analysis		Multivariable Analysis *		Propensity Score Matching	
	OR (95% CI)	*p*-Value	OR (95% CI)	*p*-Value	OR (95%CI)	*p*-Value
30-day death						
Non-ECMO	ref	<0.0001	ref	<0.0001	ref	<0.0001
ECMO	0.58 (0.54, 0.62)		0.73 (0.68, 0.79)		0.76 (0.68, 0.85)	
6-month death						
Non-ECMO	ref	<0.0001	ref	<0.0001	ref	<0.0001
ECMO	0.46 (0.42, 0.49)		0.661 (0.61, 0.72)		0.69 (0.61, 0.79)	
One-year death						
Non-ECMO	ref	<0.0001	ref	<0.0001	ref	<0.0001
ECMO	0.43 (0.39, 0.46)		0.63 (0.58, 0.69)		0.66 (0.58, 0.75)	

* adjusted for age, gender, insurance type, urbanization level, level of hospital, volume of hospital, admission route, cancer, ischemic stroke, hemorrhagic stroke, myocardial infarction, angina, heart failure, arrythmia, hypertension (with medication), diabetes mellitus (with medication), lipidemia, pulmonary disease, and chronic renal failure.

**Table 5:** Comparison of hospitalization days, hospital costs, and disposition in both groups after propensity matched analysis.

	Total	mNon-ECMO	mECMO	*p*-Value
Hospitalization days				<0.0001 ^a^
Mean ± SD *	11.1 ± 15.9	9.8 ± 15.4	12.4 ± 16.4	
Median (IQR ^†^)	4.0 (1.0–15.0)	3.0 (1.0–13.0)	6.0 (2.0–17.0)	
ICU ^‡^ days				0.0954 ^a^
Mean ± SD *	9.2 ± 19.9	10.0 ± 29.8	8.8 ± 11.2	
Median (IQR ^†^)	4.0 (2.0–12.0)	4.0 (2.0–12.0)	4.0 (2.0–12.0)	
Post-hospitalization ICU days				0.1129 ^b^
Mean ± SD *	23.8 ± 47.1	27.4 ± 53.3	21.4 ± 31.4	
Median (IQR ^†^)	11.0 (4.0–27.0)	10.0 (3.0–25.0)	11.0 (4.0–29.0)	
Hospital cost/person				
Short-term Hospital cost *				<0.0001 ^a^
Mean ± SD *	$12,017 ± 13,428	$5016 ± 7031	$19,018 ± 14,601	
Median (IQR ^†^)	$7877 (2069–17,091)	$2157 (518–6904)	$15,117 (8853–248,757)	
Long-term Hospital cost				<0.0001 ^a^
Mean ± SD *	$26,519 ± 29,592	$21,252 ± 25,548	$30,400 ± 31,714	
Median (IQR ^†^)	$17,111 (6285–35,533)	$12,780 (5063–29,686)	$20,324 (8324–42,500)	
Post-hospitalization disposition, *n* (%)				0.1299 ^c^
Continuing Admission	869 (11.4)	396 (10.4)	473 (12.4)	
Readmission	57 (0.7)	25 (0.7)	32 (0.8)	
Outpatient clinic	965 (12.6)	501 (13.1)	464 (12.1)	
No follow-up	5761 (75.3)	2904 (75.9)	2857 (74.7)	

^a^ linear regression with generalized estimating equations method; ^b^ Wilcoxon rank sum test. ^c^ symmetry test; NA is abbreviation for “Not Available”; SD *, standard deviation; IQR ^†^, interquartile range; ICU ^‡^, Intensive care unit.

## Abbreviations

aOR: Adjusted odds ratio; Absolute standardized differences; CABG, Coronary artery bypass graft; CAG, Coronary angiography; CCI, Charlson Comorbidity Index; CI, Confidence intervals; CPR, Cardiopulmonary resuscitation; CRRT, Continuous renal replacement therapy; DM, Diabetes mellitus; ECMO, Extracorporeal membrane oxygenation; ECPR, extracorporeal cardiopulmonary resuscitation; ED, Emergency department; ER, emergency room; EEG, Electroencephalography; ICD, Implanted cardioverter-defibrillator; ICU, Intensive care unit; International Classification of Diseases, 10th edition, ICD-10; Interquartile range, IQR; IHCA, In-hospital cardiac arrest; IQR, interquartile range; NHIS, National Health Insurance Services; OR, odds ratio; OHCA, Out-of-hospital cardiac arrest; OR, Odds ratios; PCI, Percutaneous coronary intervention; PS, Propensity score; SES, Socio-economic status; SD, standard deviation; TTM, targeted temperature management; QALY, Quality adjusted life year.

## Appendix A

**Table A1:** The 30-day, 6-month rate, and 1-year mortality rates before propensity score matched analysis and after propensity score matched analysis.

	Before PS Matched Analysis			After PS Matched Analysis		
	Total	Non-ECMO	ECMO	Total	mNon-ECMO	mECMO
30-day death	211,815 (83.5)	208,932 (83.6)	2883 (74.7)	5954 (77.8)	3081 (80.5)	2873 (75.1)
6-month death	228,967 (90.2)	225,840 (90.4)	3127 (81.0)	6471 (84.6)	3354 (87.7)	3117 (81.5)
1-year death	231,356 (91.2)	228,202 (91.3)	3154 (81.7)	6539 (85.5)	3395 (88.7)	3144 (82.2)

## Appendix B

**Table A2:** The comparison of demographic, hospital, and medical factors between one-year survivors and non-survivors of ECMO supported patients with cardiac arrest before PS matching analysis.

	Survivors *N* = 705 (18.3)	Non-Survivors *N* = 3154 (81.7)	*p*-Value
Age, years	55.05 ± 14.38 (705)	60.85 ± 14.83 (3155)	<0.0001
Age group, *n* (%)			
20–39 yrs	107 (15.18)	325 (10.30)	<0.0001
40–64 yrs	394 (55.89)	1374 (43.58)	
65–74 yrs	143 (20.28)	860 (27.26)	
75–84 yrs	58 (8.23)	529 (16.77)	
85~yrs	3 (0.43)	66 (2.09)	
Male	504 (71.5)	2120 (67.2)	0.0271
Insurance type, *n* (%)			
National Health insurance	683 (96.9)	2986 (94.6)	0.0073
Medical aid	20 (2.8)	167 (5.3)	
Urbanization level, *n* (%)			
Urban	627 (88.9)	2824 (89.6)	<0.0001
Rural	57 (8.09)	321 (10.17)	
Level of Hospital, *n* (%)			
Tertiary	480 (68.09)	2090 (66.24)	0.5822
Secondary	225 (31.91)	1064 (33.72)	
Volume of hospital (Beds), *n* (%)			
<300	35 (4.96)	120 (3.84)	0.0021
300–499	79 (11.21)	384 (12.17)	
500–799	277 (39.29)	1454 (46.09)	
>800	314 (44.54)	1196 (37.91)	
Admission route (ER), *n* (%)	494 (70.07)	2283 (72.39)	0.2146
Charlson comorbidity index <2	419 (59.43)	1528 (48.43)	<0.0001
Preexisting disease, *n* (%)			
Cancer	56 (7.94)	322 (10.21)	0.0676
Ischemic Stroke	57 (8.09)	390 (12.36)	0.0013
Hemorrhagic Stroke	7 (0.99)	39 (1.24)	0.5905
Myocardial infarction	41 (5.82)	218 (6.91)	0.2939
Angina	143 (20.28)	709 (22.47)	0.2052
Heart failure	48 (6.81)	347 (11.00)	0.0009
Arrhythmia	54 (7.66)	293 (9.29)	0.172
Hypertension	318 (45.11)	1689 (53.53)	<0.0001
Hypertension + medication	276 (39.15)	1372 (43.49)	0.0353
Diabetes Mellitus	189 (26.81)	1115 (35.34)	<0.0001
Diabetes Mellitus + medication	128 (18.16)	736 (23.33)	0.0029
Lipidemia	246 (34.89)	1156 (36.64)	0.3833
Chronic Pulmonary disease	130 (18.44)	676 (21.43)	0.0778
Chronic Renal Failure	21 (2.98)	223 (7.07)	<0.0001
Hemodialysis	12 (1.70)	122 (3.87)	0.0045
